# Steep Decline and Cessation in Seed Dispersal by *Myrmica rubra* Ants

**DOI:** 10.1371/journal.pone.0139365

**Published:** 2015-09-28

**Authors:** Audrey Bologna, Claire Detrain

**Affiliations:** Service d’Ecologie Sociale, Université Libre de Bruxelles (ULB), Brussels, Belgium; Indian Institute of Science, INDIA

## Abstract

Myrmecochorous diaspores bear a nutrient-rich appendage, the elaiosome, attractive to ant workers that retrieve them into the nest, detach the elaiosome and reject the seed intact. While this interaction is beneficial for the plant partner by ensuring its seed dispersal, elaiosome consumption has various effects −positive, negative or none − on ants’ demography and survival, depending on both the ant/plant species involved. In this context, the contribution of ants to seed dispersal strongly varies according to the ant/plant pairs considered. In this paper, we investigate whether the dynamics of myrmecochory also vary on a temporal scale, for a given pair of partners: *Myrmica rubra* ants and *Viola odorata* seeds. During their first encounter with seeds, ants collect all the diaspores and eat the majority of elaiosomes. Both the harvesting effort and the elaiosome consumption decline when seeds are offered on the next week and completely cease for the following weeks. This is related to a decrease in the number of foragers reaching the food source, as well as to a reduced probability for an ant contacting a seed to retrieve it. Seed retrieval is not reactivated after seven weeks without any encounter with *V*. *odorata* seeds. By contrast, naive ant colonies only fed with fruit flies do not show a decline of prey harvesting of which the speed of retrieval even increases over the successive weeks. Myrmecochory may thus be labile at the scale of a fruiting season due to the ability of ants to steeply tune and cease for several months the harvesting of these seemingly poorly rewarding items and to maintain cessation of seed exploitation. The present study emphasizes the importance of a long-lasting follow up of the myrmecochory process, to assess the stability of this ant-plant partnership and to identify mechanisms of adaptive harvesting in ants.

## Introduction

Pollination and seed dispersal are key processes in the life cycle of plants that allow them to reproduce and colonize new areas. Seed dispersal is of primary importance from an ecological [1], demographical and evolutionary point of view by ensuring genes flow between plant populations [2]. A thorough understanding of dispersal processes is thus crucial for the development of plant conservation strategies as well as for the management of invasive species [3]. Seed dispersal can be achieved by abiotic vectors such as gravity (barochory), water (hydrochory) or wind (anemochory). Thus, seeds have developed morphological and/or phenological features—called dispersal syndromes—that are adapted to the dispersal agent [4–6] such as light-weight, winged or plumed seeds in the case of anemochory [7]. Seeds can also be dispersed by biotic vectors–when seeds are accidentally or deliberately ingested and later released by defecations (endozoochory) [3]. Likewise, seeds can be incidentally attached to an animals’ body or actively transported by animals (exozoochory) [8,9].

The dispersal syndrome of myrmecochory is the presence on diaspores of a nutrient-rich appendage, the elaiosome. This elaiosome is attractive to ant foragers that collect the diaspore, bring it to the nest and thereby move it away from the mother plant. Nestmates and mostly larvae [10–15] eat the elaiosome, while the seed with its germinating potential intact is rejected from the nest in the refuse pile close to nest entrances, or even transported at longer distance inside the territory of the ant colony [8,16,17]. Ant-dispersed seeds can benefit in various ways: rejection into nutrient-enriched microsites, reduction of siblings/parent-offspring plant competition [18], reduced exposure to seed predators, fire avoidance [19], germination facilitation [20] or broken dormancy induced by ant chewing behaviour [11].

The attractiveness of the diaspores (the seed and its elaiosome) to ant foragers–and thus the dispersal efficiency–depends on several attributes, such as the size of the elaiosome [8,9,21,22], the elaiosome/seed ratio [13,23] or the chemical compounds found in elaiosomes [24–26]. As myrmecochory is a case of active exozoochory, the fate of dispersed seeds depends on the traits of both the seed and the elaiosome, but is also closely related to the ants’ foraging behaviour. In this respect, ants are well known to adapt their foraging behaviour according to the value of the food, such as its nutritive content [27–29], its amount [30], or its spatial distribution [28,31,32].

In the case of myrmecochory, the nutritional value derived by ants from foraging and consuming seed elaiosomes is rather controversial. The elaiosome consumption can be beneficial to ants by increasing the weight of larvae [33], as well as the weight of sexual females that feed on elaiosomes [34] or by shifting the sex ratio towards queen production [35,36]. On the other hand, it may have no effect on ant colonies’ demography [15] or even a negative impact by slowing down brood development [37]. This insect-plant interaction can thus be asymmetrical for some ant-seed pairs [38], with unequal gains or even detrimental outcomes for the ants. In the latter case, the ant partner may hypothetically shift its foraging to other available food resources, as well as alternative partnership. Furthermore, even in the absence of obvious detrimental effects of elaiosome consumption, ants are expected to tune the intensity of seed harvesting over time when the time and energy investment spent in the retrieval of relatively heavy diaspores or in the hard cutting-off of the elaiosome is not compensated by a sufficient nutritive return.

A cessation of harvesting of myrmecochorous seeds has already been observed [39] on a short time scale, by giving diaspores *ad libitum* during a few hours. This phenomenon persisted for as long as one week and was explained by a limitation of space available inside the nest, which prevented further seed retrieval, as well as by a satiation. However, such a cessation may reflect a more fundamental phenomenon–the inherent lability of the link between some ant and plant partners involved in myrmecochory.

In this respect, little is currently known about how ants maintain their partnership with ant-dispersed seeds and possibly adjust their harvesting behaviour over the time scale of a fruiting season (several weeks), independently of any satiation effects. Therefore, in this paper, we investigate the temporal evolution of myrmecochory when *Myrmica rubra* ants are offered sweet violet diaspores (*Viola odorata*) for 5 successive weeks, which is a timescale similar to that of *V*. *odorata* seed availability in nature. To exclude any effect of nutritional satiation, only 20 diaspores were offered once per week. We investigated whether changes in the dynamics of retrieval by ants, that are mainly carnivorous, are specific to myrmecochorous seeds or common to other small food items by offering fruit flies to naïve *M*. *rubra* colonies, following the same experimental procedure as for seeds. Fruit flies are similar to diaspores in size as well as to elaiosomes in their fatty acid composition [24]. However, unlike diaspores of which ants have to carry an additional weight of unpalatable material (the seed), fruit flies can be nearly completely eaten and are undoubtedly a source of proteins beneficial to ants. Finally, by carrying out this study in controlled laboratory conditions, we were able to perform observations that couple over a long time scale the ant foraging behaviour outside the nest, to the management of retrieved seeds inside the nest.

## Materials and Methods

### Ant species: collection and laboratory rearing

*Myrmica rubra* (Linnaeus, 1758) is a common ant species in North temperate areas [40]. They form polygynous colonies of hundreds of workers [41], being 4 to 5 mm long, and queens around 7 mm [42]. While its diet is mainly carnivorous [43], this species regularly forages on sugar sources [44], such as extra-floral nectar or honeydew [45] and occasionally retrieves elaiosome-bearing seeds [46].

This red ant species has been used in previous studies as a model for the study of myrmecochory in temperate habitats [21,47,48]. Furthermore, it has recently been identified as a ‘keystone species’ due to its prevailing role as seed dispersing agent compared to other local ant species [49].

The 13 colonies tested with diaspores (test colonies) were collected during summer 2011 and 2012 on several sites in Belgium: Udange (49°38’10.776”N, 5°46’14.63”E), Chièvres (50°33’34.394”N, 3°44’39.339”E) and Gembloux (50°33’47.988”N, 4°41’38.003”E). The 8 colonies tested in the control experiment (control colonies) with *Drosophila melanogaster* prey were collected during summer 2014 on several sites in Belgium: Udange (49°38’10.776”N, 5°46’14.63”E), Crupet (50°20'47''N, 4°57'8''E) and Falisolle (50°25’10.992”N, 4°37’50.015”E). No specific permissions were required for these locations/activities. The field studies did not involve endangered or protected species. All colonies contained brood covering areas of similar size inside the nest, 300–800 individuals and 3–9 queens. In the laboratory, colonies were reared in plaster arenas (50 × 35 × 10 cm) with Fluon-coated borders to prevent ants from escaping. The nest was delimited by microscope slides covered by a glass plate with a red plastic film above (Janet type- nest 15 × 15 × 0.4 cm) perforated in its center with a hole (diameter of 1.5 cm) that allowed us to take out of the nest the seeds that were retrieved by the ants.

Colonies were regularly moistened with water (3 times per week) and were kept at a room temperature, between 18°C and 21°C, a relative humidity of 60% to 65% and a constant photoperiod of 12h per day. Ants were supplied with sucrose solution (0.3 M) *ad libitum* and one *Tenebrio molitor* mealworm three times a week.

### Plant species: seed collection and storage

The sweet violet, *Viola odorata* (Linnaeus, 1758), is a perennial herbaceous plant that is common in temperate areas like forests, edge bushes, open or ruderal habitats [50]. This plant blooms in spring, from March to May. *V*. *odorata* is an obligate myrmecochorous species, meaning that its seeds are dispersed only by ants without any previous ballistic release. This species can also rely on vegetative multiplication. Seeds are yellow-brown, about 2.5 mm in length and 2 mm in width. The white and fleshy elaiosome is smaller than the seed (about 2 mm length and 1.5 mm width).

Mature seeds of *V*. *odorata* were collected in June 2011 on the university campus (Brussels) and immediately stored at -20°C until being used for experiments. This storage procedure was shown not to influence seed attractiveness nor their removal by ants [21].

Fruits flies (*Drosophila melanogaster*) used for control colonies were stored in the same way as *V*. *odorata* seeds.

### Experimental procedure

**Fig 1:**
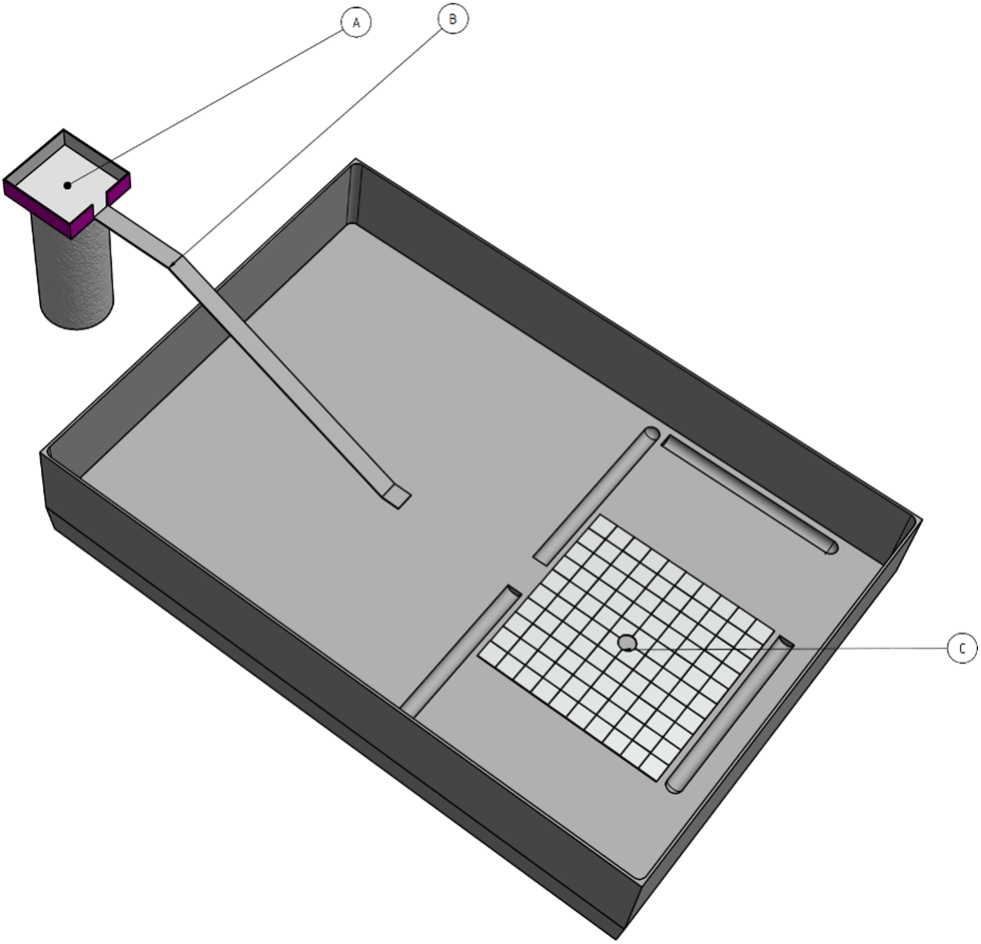
Experimental setup. The experimental setup consists of (a) a foraging platform (b) a bridge and (c) the nest.

**Fig 2:**
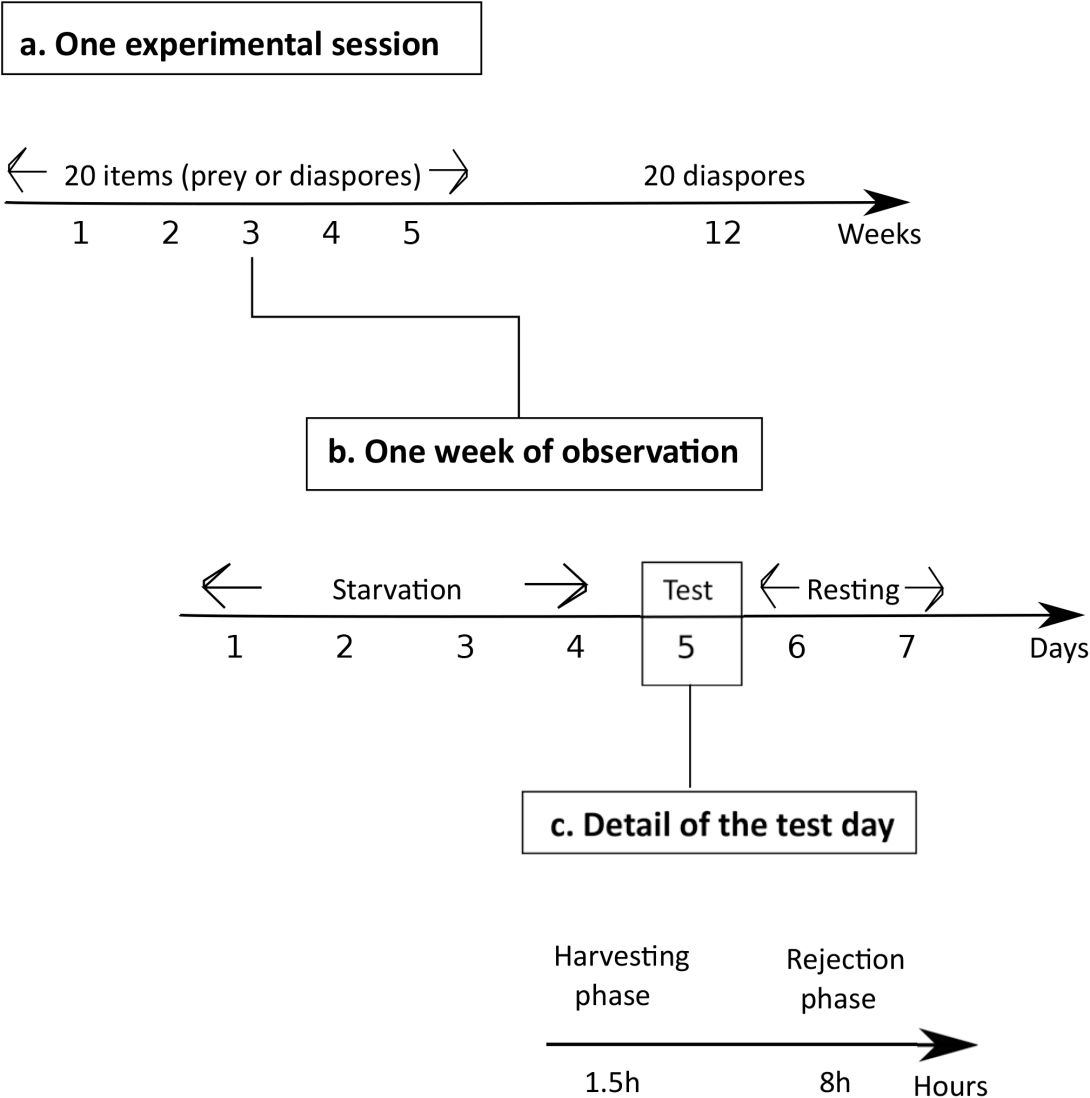
Summary of the experimental procedure. Only the « Test Colonies » have a 12th week of exposure, not the « Control Colonies ».

The experimental setup (Fig 1) consisted of a bridge that connected the nest arena to a foraging platform upon which the items–seeds or prey- were offered during the test. Each colony was tested once a week with 20 food items, during 5 successive weeks, the whole constituting one experimental session (Fig 2A). Some colonies (“Test Colonies”, N = 13) were faced exclusively with seed while others exclusively with prey (“Control Colonies”, N = 8).

Each week of observation was divided into: 1°) a starvation (D1 to D4) and familiarization period (D4), 2°) the test day (D5) and finally 3°) a resting period (D6 and D7) (Fig 2B). The colony was first starved for four days by removing any food from the nest. On the 4^th^ day, the colony was allowed to familiarize with the experimental setup by connecting the nest to a foraging platform with a bridge. On the 5^th^ day- i.e. the test day- the colony was offered food on this platform: either 20 *V*. *odorata* seeds (during 5 weeks and at week 12) or 20 fruit flies (during 5 weeks). The last two days consisted in a resting period during which colonies were supplied with sucrose solution (0.3 M) *ad libitum* and one mealworm before being starved again.

During the test day (D5), we observed diaspores/prey harvesting during 90 minutes as well as elaiosome/prey consumption inside the nest. At the end of this 90 minutes period, the bridge was removed to observe the dynamics of seed rejection outside the nest for 8 hours (Fig 2C). A long-term monitoring was achieved for 11 among the 13 “Test Colonies” for which foragers were no longer exposed to myrmecochorous seeds during 7 weeks and were again offered *V*. *odorata* diaspores at week 12. During the harvesting phase, we counted the number of items remaining on the foraging platform every five minutes. We measured the flow of foragers arriving on the foraging platform every five minutes during the first 30 minutes as long as items were still available on the foraging area.

For the “Test Colonies”, we calculated the probability for an ant to take a seed by counting the number of contacts before deciding to retrieve it. This was averaged over the three first foragers reaching the platform. Then, colony values were averaged over the 13 tested colonies.

Inside the nest, an index of interest towards each type of item was defined as the mean number of inner-nest ants actively discarding elaiosomes or eating insect prey. It was calculated both for the “Test Colonies” and for the “Control Colonies”. This index of interest was measured as soon as the first item entered in the nest (around 15 minutes after the beginning of the experiment) and counted every 15 minutes until the end of the experiment.

During the rejection phase, we counted every 30 minutes the number of seeds removed out of the nest and we noted whether they still bore an elaiosome (“Test Colonies”). For “Control Colonies”, we checked any rejection of whole prey after 8 hours.

### Statistical analysis

To test the impact of “Week” and “Colony” factors on the removal of diaspores/prey from the foraging platform, we used the Cox proportional hazard model [51]. In comparison with other survival models such as parametric ones, this Cox model does not make any specific assumption on the statistical distribution of the hazard [52]. The adequacy of the Cox model for our data was assessed following the method of Bradburn and colleagues [53]. Then for the only factor having a significant effect on seed removal (“Week”), we checked when the retrieval dynamics became statistically different from that observed on the first exposure to seeds (taken as the reference week) by using the log-rank test for each colony [51,54].

The number of diaspores left at the foraging platform after 90 minutes was compared between the weeks 1 to week 12-for the 11 colonies having a long-term monitoring-by using the Friedman test followed by a Nemenyi post-hoc test.

For each colony of the “Test group” and for each week, the cumulative flow of ants reaching the foraging platform was best-fitted by a straight line, with a R^2^ comprised between 0.99 and 0.83 (except for two fittings with a R^2^ equal to 0.5 and 0.6 when there was a too weak flow of ants of less than 10 ants in total). The slopes of linear fittings that correspond to the arrival rates of foragers per minute were compared by using a Friedman test followed by a Nemenyi post-hoc test This was made on 12 colonies, a technical problem leading to missing values for one colony at week 4.

The interest index of inner-ants towards seeds or fruit flies compared between the 1^st^ and the 2^nd^ exposure using paired t-test. Differences in the proportion of seeds rejected with or without elaiosomes across weeks were analyzed for statistical significance by using Chi-square test.

To assess the quantity consumed by ants, we multiplied the number of elaiosomes or insect prey eaten by the mean weight of defrosted fresh food. We weighed five batches of five defrosted elaiosomes each (15.6 ± 4 mg, N = 5), as well as five batches of five defrosted insect prey each (39.6 ± 4 mg, N = 5), allowing us to approximate the weight of one elaiosome (3 mg) as well as the weight of one insect prey (8 mg).

Statistical analyses were made using R software version 2.1.0 and survival analyses were performed using the “survival” package for R. All statistical tests were two tailed with a significance threshold of 0.05. All the mean values are given as the average ± the standard error.

## Results

**Fig 3:**
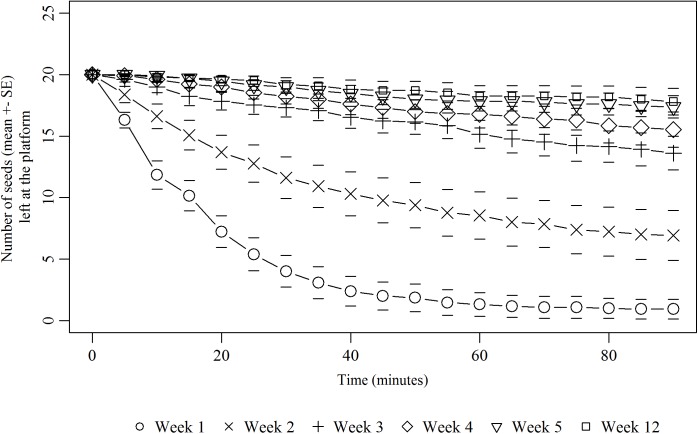
Removal dynamics of *Viola odorata* diaspores during the five exposures. Dynamics of removal of *V*. *odorata* diaspores by ants (mean ± SE) at the foraging platform during the harvesting phase (90 minutes) for 5 successive weeks (Week 1 to Week 5, N = 13) and at after 7 weeks without any exposure to seeds (week 12, N = 11).

While seed retrieval by ants did not show any inter colonial differences (PH Cox model, N = 1300, Z = -0.529, p>0.05), it declined across the five successive weeks of exposure (PH Cox model, N = 1300, Z = -22.270, p<0.01). On the first exposure (week 1), all seeds were harvested from the foraging platform after 60 minutes for most of the colonies (Fig 3). Then, on the 2^nd^ seed exposure, the harvesting process started to slow down. This decline in seed harvesting was significant for 9 colonies (W_1_ vs W_2_: log-rank test on survival curves of each colony with p-value <0.05) amongst the 13 colonies tested. At the 3^rd^ exposure, this decrease became more pronounced and significant for all colonies tested (W_2_ vs W_3_: log-rank tests, all p-values <0.05). Even though ants reached the foraging platform and contacted seeds, almost no seed was taken back to the nest. Such a decrease persisted for the next two exposures for all colonies, and became even more pronounced for 3 colonies at week 4 (W_3_ vs W_4_: log-rank test, all p-values <0.05) and 2 colonies at week 5 (W_4_ vs W_5_: log-rank test, all p-values <0.0001). Furthermore, for the 11 colonies that were tested on the long term, workers that contacted diaspores at week 12, still did not retrieve them, despite that those colonies did not encounter *V*. *odorata* seeds nor feed on their elaiosome for nearly two months. Seed harvesting activity thus remained extinct for several months and was similar to the ones observed previously on weeks 3 to 5 (Friedman test, N = 6, W_1_ to W_5_ and W_12_, df = 5, χ^2^_r_ = 40.34, p<0.0001; Nemenyi post-hoc test, W_12_ = W_3_, W_4_ and W_5_).

**Fig 4:**
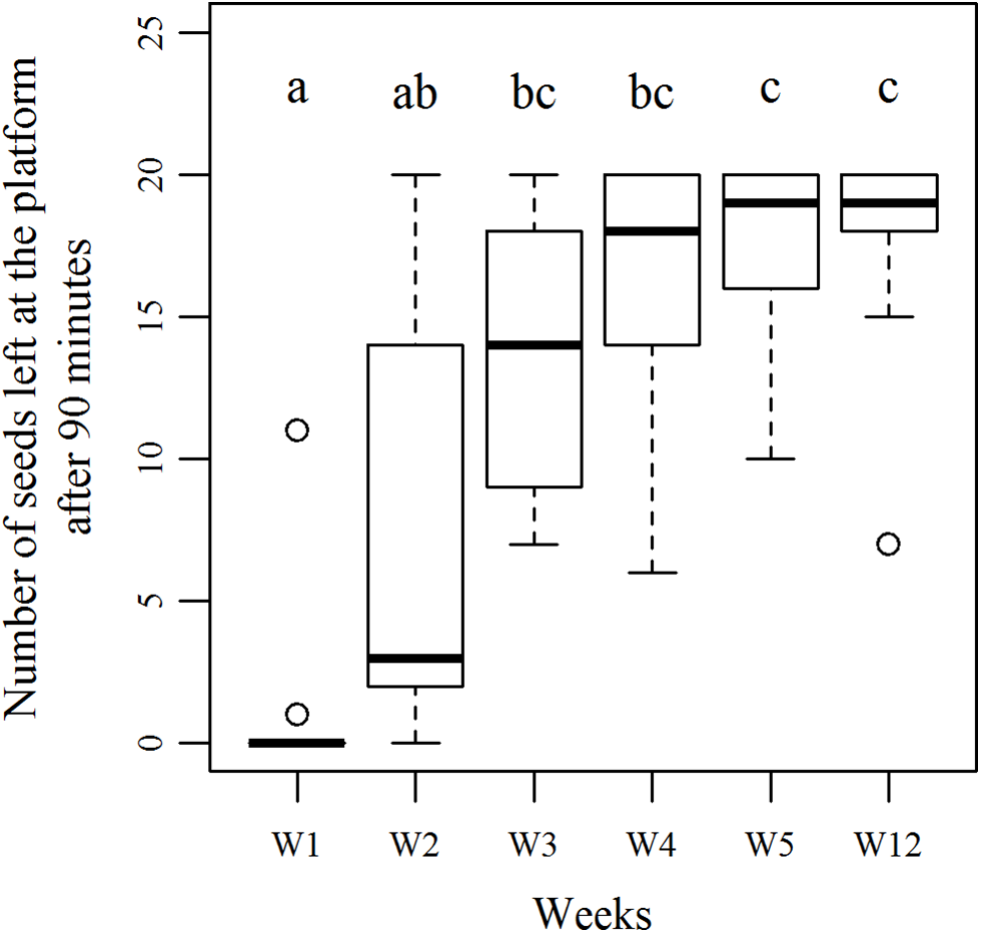
Number of *V*. *odorata* seeds left on the foraging platform at the end of the harvesting phase for week 1 to week 5 as well as week 12. N = 11 for each week. Boxes show lower and upper quartile and the median, whiskers assign minimum and maximum values and plus outliers. Boxes with the same letters are not significantly different (Friedman test followed by a Nemenyi post-hoc test).

As a result of this cessation process, the median number of seeds that was ultimately left on the foraging platform significantly increased along successive exposures to the same ant colony (Fig 4.) (Friedman test, N = 5, W_1_ to W_5_, df = 4, χ^2^_r_ = 39.85, p<0.0001). On the first exposure at week 1, no seed was left on the foraging platform for 10 colonies amongst the 11 tested on the long-term. At the 2^nd^ exposure, the number of non-retrieved seeds increased but was not significantly different from the 1^st^ exposure (Nemenyi post-hoc test, W_1_ = W_2_) due to the high inter colonial variability of harvesting effort. From the 3rd seed exposure onwards, the number of seeds left at the foraging platform significantly increased compared to W_1_ (Nemenyi post-hoc test, W_1_≠W_3_-W_5_).

As regards the mobilization of foragers, we measured the number of ants arriving at the platform as long as seeds were still available. For each colony and for each exposure to seeds, the cumulative number of ants that were mobilized to the foraging area grew in a linear way (Linear regression, N = 58, R^2^ values ranging between 0.99 and 0.85), which indicates that there was no active recruitment of foragers towards the seed patch even when first encountered. This was confirmed by the absence of ants that were seen laying a trail.

**Fig 5:**
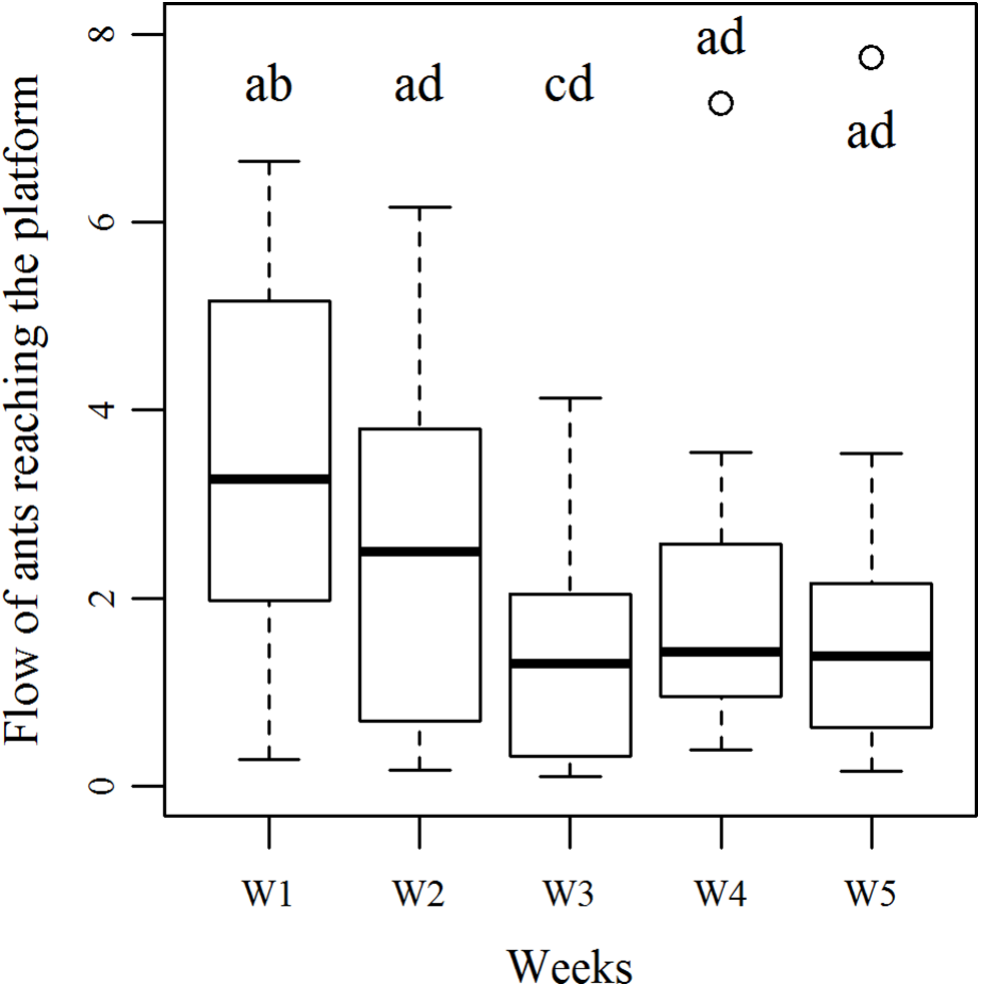
Number of ants reaching the platform. Number of ants arriving on the foraging platform every minute (N = 12), during the first 30 minutes of the harvesting phase (when there were still seeds available). Boxes show lower and upper quartile, median, whiskers assign minimum and maximum values and (circles) outliers. Boxes with the same letters are not significantly different (Friedman test followed by a Nemenyi post-hoc test).

The flow of foragers per minute (Fig 5) changed across exposures (Friedman test, N = 5, df = 4, χ^2^_r_ = 15.85, p<0.05). Indeed, while ants’ flow at week 2 was similar to that first observed at week 1 (Nemenyi post-hoc test, W_1_ = W_2_), it significantly decreased of almost one third at exposure 3 (Nemenyi post-hoc test, W_1_≠W_3_). During the last three exposures (week 3, week 4 and week 5), the flow of ants did not change anymore and remained quite low (Nemenyi post-hoc test, W_3_ = W_4_ = W_5_,).

In addition to a decreasing number of ants reaching the foraging platform (Fig 5), we observed a behavioural change of foragers that became less likely to take a diaspore back to the nest from week 1 to week 5. This has been calculates for the weeks in which the change in seed harvesting was highly significant–i.e: week 1, week 2 and week 5. While 33% of ants retrieved a seed after contacting it at week 1 (N = 38), this percentage decreased to 23% at week 2 (N = 37) and was even lower at week 5 (10%, N = 35). Amongst those ants that brought seeds back to the nest, the number of contacts needed to trigger seed transport increased from week 1 to week 2. While one contact was sufficient to trigger seed removal by 60% of foragers at week 1 (N = 13), at least two contacts were required at week 2 (N = 9).

**Fig 6:**
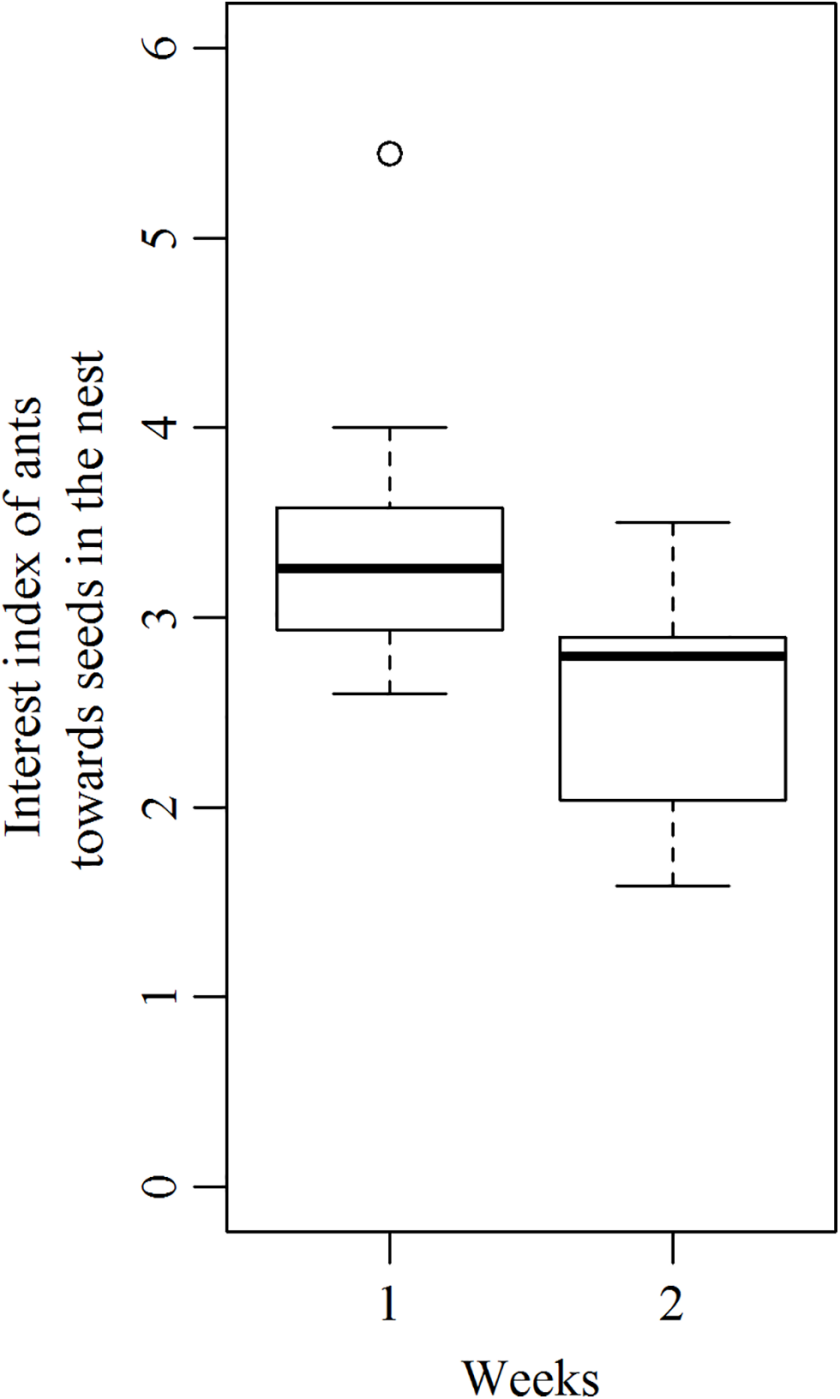
Interest Index of workers towards seeds inside the nest. This has been measured for week 1 and week 2 for which a sufficient number of seeds (at least 3) were brought back to the nest. N = 10.

Concerning the management of seeds inside the nest, there was a progressive disinterest of workers towards diaspores. This was observed from week 1 to week 2 when seeds were still taken by foragers and for 10 colonies (out of the 13 tested ones) that retrieved at least 3 seeds inside their nest. On average, 3.3 ± 0.82, ($\bar{\text{x}}$+SD; N = 10) ants surrounded diaspores and were involved into the removal of seed elaiosome by actively chewing it when first encountering seeds. However a significantly lower number of 2.80 ± 0.59, (N = 10) workers did this task on the next exposure to seeds (Fig 6 Paired Student test, t = 2.63, p<0.05).

**Fig 7:**
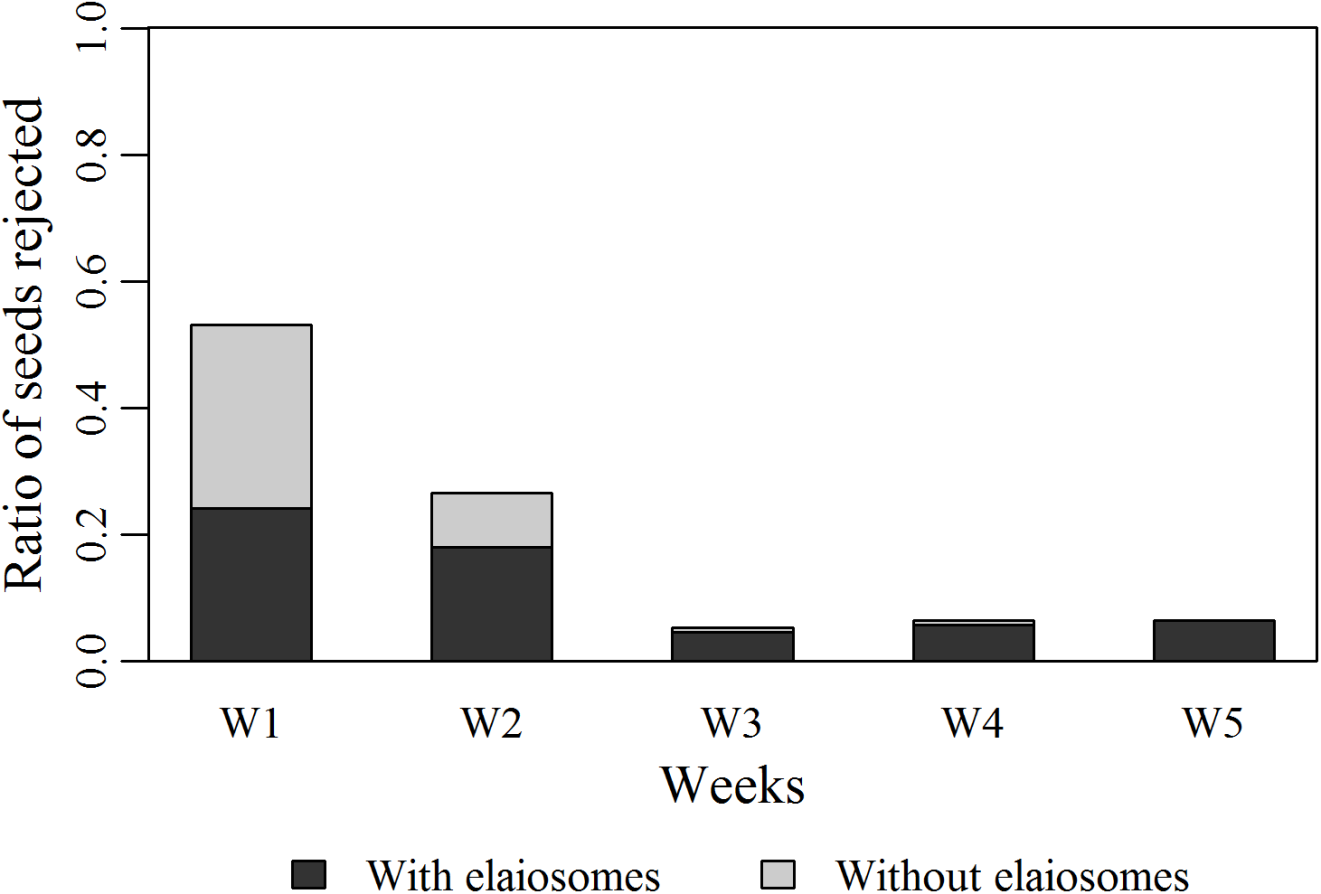
Seeds rejection outside the nest. Ratio of seeds rejected after 8 hours amongst all seeds offered (13 x 20, N = 260) for each seed exposure. Rejected seeds were divided into two categories according to their state: with or without elaiosome.

Concerning the rejection from the nest, after 8 hours, it decreased on average from 12 ± 5 seeds ($\bar{\text{x}}$+SD; N = 13) for the first exposure to 6 ± 3 seeds (N = 11) for the second exposure to diaspores (Mann-Whitney test, U = 19.5, p = 0.0027). Amongst all seeds offered (N = 260 each week in total for all colonies), the ratio of seeds rejected after 8 hours was of 0.53 on the first week (Fig 7) and decreased by half on week 2 with a ratio of 0.26 (Fig 7). This decrease in the ratio of seeds rejected-and then ultimately dispersed by ants- is mainly due to the cessation of seed harvesting. During the following weeks, a small amount of seeds were retrieved inside the nest but the majority of them were released after 8 hours.

Furthermore, among the total number of seeds rejected outside the nest, the proportion of elaiosomes actually consumed decreased significantly across exposures until a complete stopping at week 5 (Fig 7; Chi-square test, χ^2^ = 31.67 p<0.0001). The cessation of ants’activity thus occurred not only for the harvesting of seeds but also for the consumption of their elaiosome. During the first exposure to *V*.*odorata* seeds, each ant colony consumed a mean of 17 ± 16 mg ($\bar{\text{x}}$+SD; N = 13) out of the 60 mg of offered elaiosomes. This quantity decreased during the following weeks with no more than 5 ± 8 mg (N = 13) of elaisomes being consumed by ants, to none being eaten from the third week onwards.

Using the same experimental procedure as for diaspores, we offered dead fruit flies to eight *M*. *rubra* colonies to highlight a possible cessation of retrieval of these food items due to a weekly repeated exposure.

**Fig 8:**
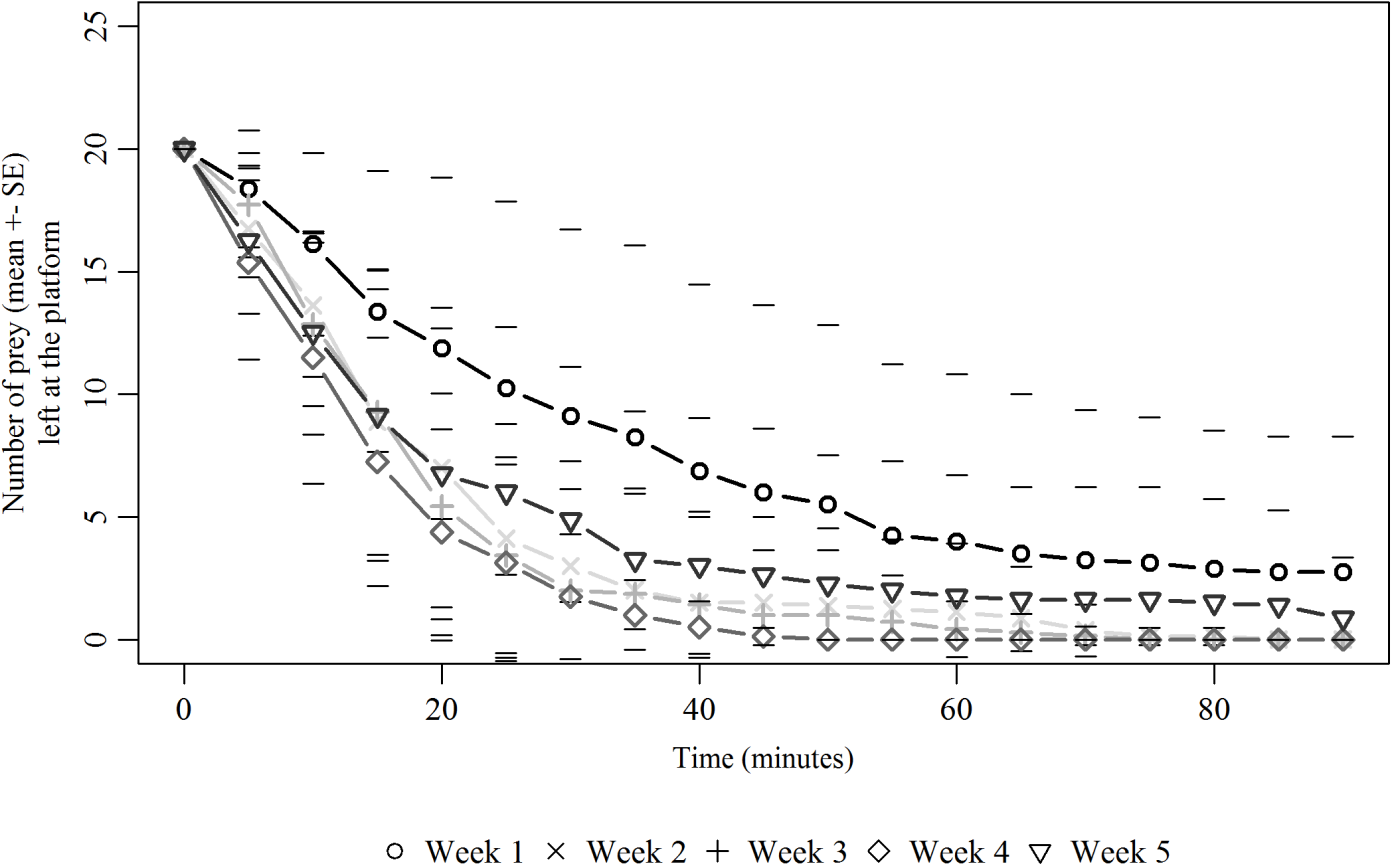
Removal of *Drosophila melanogaster* prey during the five successive exposures. Dynamics of removal of *D*. *melanogaster* prey by ants (mean ± SE) at the foraging platform during the harvesting phase (90 minutes) for 5 successive weeks (Week 1 to Week 5, N = 8).

Dynamics of prey removal from the foraging platform differed between colonies (PH Cox model, N = 780,Z = 6.217, p<0.05) as well as across the five successive weeks (PH Cox model, N = 780, Z = 8.136, p<0.05). At the first week of exposure, a few prey still remained at the foraging platform at the end of the experiment (Fig 8). Over the following weeks (week 2 to week 5), ants brought all the prey items back to the nest and furthermore, increased their removal speed. Thus, the 5-weeks time evolution of foraging on fruit flies strongly differed from that of seed harvesting. Instead of declining as for *V*. *odorata* diaspores, there was a slight acceleration of fruit flies retrieval towards the nest. Regarding the number of non-retrieved prey items, at the first exposure, only 2 colonies (out of the 8 tested ones) did not remove all the prey and left respectively 7 and 15 *D*. *melanogaster* on the platform at the end of the experiment. During the following weeks (week 2 to week 4), all the prey were removed and brought back to the nest for all colonies (excepting one due to the fewer number of foragers exploring the food location).

**Fig 9:**
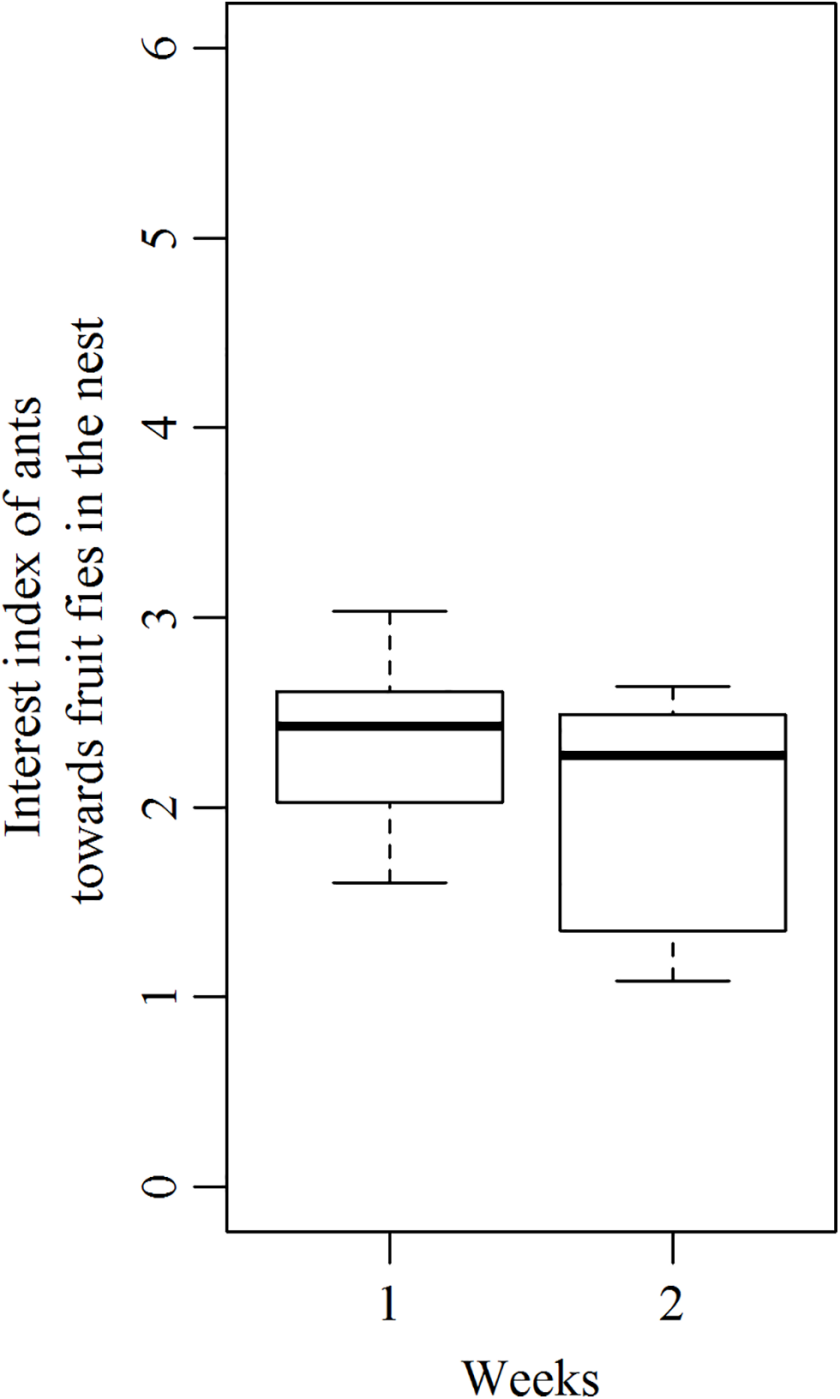
Interest Index of workers for insect prey inside the nest for exposures 1 and 2. N = 8.

Colonies that were offered prey showed an interest index that did not change from week 1 to week 2 (Fig 9 (Paired Student test, t = 1.85,p>0.05)). Indeed, an average of 2.34 ±0.46 ants ($\bar{\text{x}}$+SD; N = 8) actively consumed insect prey inside the nest at week 1 and this number did not change significantly at week 2, with 1.99± 0.64 ants (N = 8) around insect prey. Moreover, during the 5 successive weeks, we did not observe any rejection of prey outside the nests since they were all totally consumed what corresponded to a total of 160 mg of food consumed per colony per week, which is quite high compared to the quantity of elaiosomes consumed by the test colonies.

## Discussion

We show that, the exploitation of *V*. *odorata* diaspores by *M*. *rubra* ants rely on individual retrieval behaviour, without any nestmates’ recruitment. Faced with the same myrmecochorous seed species during 5 successive weeks, *M*. *rubra* ants first steeply decrease and then completely cease the exploitation of those seeds through time. The loss of interest towards those diaspores occurs at all steps of the myrmecochory process.

First, on the foraging platform, fewer diaspores are retrieved across exposures due to a concurrent decrease in the flow of foragers as well as in their individual probability to collect a seed. Second, inside the colony, the interest of workers towards diaspores–in particular to elaiosomes–strongly declines. There is thus a progressive loosening of this ant-plant interaction with a concurrent decrease in the number of seeds dispersed as well as in the elaiosome consumption. This cessation of foraging was shown to persist over the long-term for at least 2 months.

A first hypothesis to account for a decrease of seed harvesting by ants could be a satiation effect that would occur if ant colonies had collected all the food that it needed for several weeks or that it was able to process and store within the nest. However, this is quite unlikely in our case. First, *M*. *rubra* colonies that are formed of several hundreds of workers and larvae retrieved only a small number of *V*.*odorata* seed items, among which just a few elaiosomes (at most 25% of all the offered seeds) are removed exclusively during the first retrieval events and for which little elaiosome amount is eaten with no more than 22 mg being consumed per colony for two months. Furthermore, when fruit flies are offered during several weeks, colonies do not lose their interest for insect prey inside the nest or do not show a decrease but instead an acceleration of their foraging activity. This confirms that ant colonies can forage for several weeks over an amount of food items (160 mg preys per week during 5 weeks) that is far larger than that of retrieved elaiosomes, without showing any satiation effect. Second, there is no evidence for a congestion of the chain of seed processing due to a lower food demand or to a lack of space. Indeed, during the successive weeks of observations the brood amount–in terms of the area occupied in the nest- remained unchanged, and enough nest space was always available for seed storage.

A second hypothesis is that the cessation of diaspore harvesting, as well as elaiosome consumption, results from avoidance learning. Avoidance learning is a well-known process occurring in social insects such as honey bees [55] or ants [56–58].

For example, in the context of ant-plant relationship, foragers of leaf-cutting ants [59,60] are able to associate olfactory cues to leaves containing toxic compounds harmful for their fungus, and thereby to learn to avoid retrieving this food material [56]. Likewise, extinction towards plant material treated with a fungicidal agent or organic volatile compounds (VOC) occurred in *Atta* and *Acromyrmex* leaf-cutting ants and persisted on the long term, up to 30 weeks [57,61,62]. Likewise in the case of myrmecochory, elaiosomes are meant to attract ant species for seed dispersal [24], but may also concurrently help avoiding granivory by repelling seed predators with secondary toxic metabolites. Such a “dual function”–attracting mutualists and repelling seed predators- has been observed for one myrmecochorous species, *Corydalis aurea*, of which elaiosomes contain alkaloids that repel *Peromyscus maniculatus* rodents [63]. This “dual function” is also well-known for plant nectars in which sugars attract pollinators or defenders against herbivores while secondary metabolites and volatile compounds repel nectar robbers or prevent the growth of pathogens; review in: [64].

A third explanation is that *M*. *rubra* workers can finely tune their foraging activity through time, based on the expected weak benefits drawn from elaiosome consumption compared to the costs associated to diaspore retrieval and dispersal. Ants may have perceived this weak return either as a too low nutritional quality of elaiosomes or as a too high energetic/time investment to transport heavy seeds and/or to remove elaiosomes. A cessation of food plant consumption due to a low nutritional content has already been observed for several insects for instance in the grasshopper *Schistocerca americana* nymphs [65]. In the latter, an internal feedback about food suboptimality-in terms of sterols’ availability- is responsible for the interruption of feeding on spinach leaves [66]. Ants are also able to adapt their foraging activity when they are faced with unbalanced diets [27,67,68] and to even considerably reduce the exploitation of such diets for at least 18 days [27]. Then, it would be worthwhile to investigate whether the cessation of seed collection and elaiosome consumption varies between plant species according to their chemical content and expected nutritional value of elaiosomes.

The rapid cessation of harvesting may even point to cheating rather than reward on behalf of the plant partner [26,37,69]. Indeed, compounds such as oleic acid [26,37,70]- or the polar lipid fraction [69]- found in elaiosomes trigger seed retrieval by ants by mimicking the haemolymph of an insect prey in its fatty acid composition [24], independently of their actual nutritive value. Thereby, elaiosome-bearing seeds could act as a “sensory-trap” since they are mimics of honest signals that elicit responses of the receiver for out-of-context goals of the signal emitter [71].

Regardless of the underlying behavioural mechanisms involved, the cessation phenomenon of *V*. *odorata* seed harvesting by *M*. *rubra* ants appears as a new testimony of the ability of foragers to adapt their individual and collective response to actual food rewards [28,72–76]. The magnitude of cessation in myrmecochorous seed dispersal has still to be assessed by investigating other ant-plant partnerships. Indeed, in a diffuse mutualism such as myrmecochory, there are several plant species of which seeds can be dispersed by ants. In its turn, each of these diaspores can be attractive to several ant species belonging to the guild of seed dispersers [49,77]. There is thus a wide range of possible partnerships of which the strength may deeply vary through time and may be influenced by the cost/benefit balance gained by the ants.

The so far neglected stability of myrmecochory undoubtedly raises interesting ecological and evolutionary issues. The observed cessation of myrmecochorous seed harvesting reveals a lack of stability of this ant-plant relationship. In our observations, ants decrease their dispersal service until complete seed avoidance for at least 2 months. This seems to be highly prejudicial for myrmecochorous plants of which seed dispersal by ants would be time limited [39]. Several factors however may soften the detrimental effects that follow the cessation of harvesting and may contribute to sustain myrmecochorous strategies of seed dispersal in plants. Firstly, several myrmecochorous plants benefit from alternative dispersal strategies [8,78–80] such as vegetative reproduction as it is the case for the sweet violet. Secondly, there is a yearly replacement of ant foragers after the diapause period, meaning that mostly naive individuals encounter diaspores, and thus retrieve seeds in early spring [81,82]. Thirdly, myrmecochory is a diffuse mutualism (for example: [13,49,83,84]), meaning that plants can rely on other ant colonies from the same or different species to disperse their seeds. In the case of *Myrmica spp* species living in grasslands, their foraging territories are overlapping those of other seed dispersing ants that could then exploit the same plant although at different time of the day. Furthermore, due to the polydomous structure of *M*. *rubra* colonies [42,85] each nest subunit that exploits the same plant independently, may experience a cessation of seed harvesting at different time of the fruiting season, what may contribute to the maintenance of myrmecochory. Fourthly, strictly myrmecochorous plants usually maximize their chance to be dispersed by ants [86,87] either by releasing their seeds in early spring [46,70], when alternative resources (e.g. insect preys) are still scarce for the ants that have to focus their foraging on elaiosomes [48,88], by releasing diaspores when there are daily peaks of activity for the most effective ant dispersers [86], or by releasing seeds during a fruiting period that is shorter than the time needed for avoidance learning by ants.

Finally, a cessation of seed dispersal by ants at the scale of a fruiting season does not necessarily imply that this ant-plant interaction is unstable at the time scale of coevolution. A mildly attractive diaspore might be an evolutionary stable strategy for the plant. Naive colonies would collect seeds and then adjust their foraging behaviour according to the cost/benefit ratio of collecting these diaspores as predicted by the optimal foraging strategy [89–91]. The amount of seeds dispersed during the learning phase might then compensate for the limited cost of producing such an elaiosome. If the plant would produce a richer/bigger elaiosome in order to keep ants collecting diaspores for a longer time, the benefit in terms of seed dispersal might no longer compensate the higher energetic cost of producing a richer/bigger elaiosome.

Our study confirms that mutualisms are complex and highly dynamic interactions, in which the links between partners are not fixed but in constant evolution depending on their respective benefits. Research should now be carried out to assess the occurrence of such a cessation phenomenon for other ant-plant species, to investigate the mechanisms involved, as well as to identify the main actors–foragers, nurses and/or larvae responsible for the cessation of this ant-plant interaction. Finally, the surprisingly persistence of this cessation phenomenon over several months raises new questions on how a long-term memory of poor food quality and its resulting avoidance can be maintained at an individual and/or collective level.
